# SAPs as a new model to probe the pathway of centriole and centrosome assembly

**DOI:** 10.1042/BST20200833

**Published:** 2021-05-07

**Authors:** Alan Wainman

**Affiliations:** Sir William Dunn School of Pathology, University of Oxford, South Parks Road, Oxford, OX1 3RE, U.K.

**Keywords:** biological models, centrosomes, *Drosophila melanogaster*

## Abstract

Centrioles are important cellular organelles involved in the formation of both cilia and centrosomes. It is therefore not surprising that their dysfunction may lead to a variety of human pathologies. Studies have identified a conserved pathway of proteins required for centriole formation, and investigations using the embryo of the fruit fly *Drosophila melanogaster* have been crucial in elucidating their dynamics. However, a full understanding of how these components interact has been hampered by the total absence of centrioles in null mutant backgrounds for any of these core centriole factors. Here, I review our recent work describing a new model for investigating these interactions in the absence of bona fide centrioles. Sas-6 Ana2 Particles (SAPs) form when two core centriole factors, Sas-6 and Ana2, are co-over-expressed in fruit fly eggs. Crucially, they form even in eggs lacking other core centriole proteins. I review our characterisation of SAPs, and provide one example of how they have been used to investigate the role of a core centriole protein in PCM formation. I then consider some of the strengths and weaknesses of the SAP model, and discuss them in the context of other models for centriole study in *Drosophila*. Similar aggregates have been seen in other systems upon expression of centriole factors, so SAPs may also be a useful approach to study centriole proteins in other organisms.

## Introducing centrioles and centrosomes

**Figure 1.**
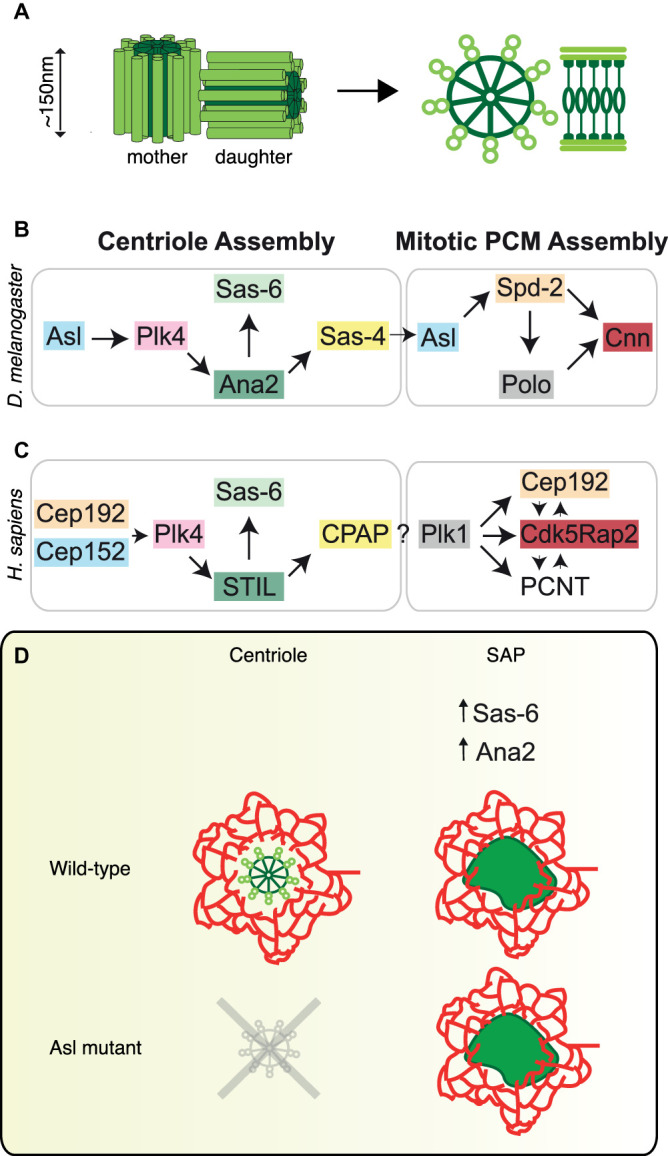
Centriole and SAP formation. (**A**) Cartoon depicting mother and daughter centrioles from *Drosophila* embryos, showing a central, 9-fold, cartwheel (dark green) surrounded by doublet MTs (light green). (**B**). In *Drosophila*, centriole duplication requires five essential proteins (Plk4, Sas-6, Ana2, Sas-4 and Asl) and mitotic PCM formation requires three proteins (Polo, Spd-2 and Cnn). (**C**) These components and the pathway for centriole formation is well conserved in humans. The coloured boxes in **B** and **C** represent homologous proteins. Both sequence and function appear to be highly conserved throughout most higher eukaryotes. (**D**) SAPs, formed in eggs when Sas-6 and Ana2 are co-over-expressed to moderate levels, can be used to probe the PCM formation pathway in the absence of bona fide centrioles. In the example described here, from our recent paper [41], we used SAPs to show that the PCM can form, albeit inefficiently, in the absence of Asl.

Centrioles are cylindrical-shaped organelles, formed by a central cartwheel with a 9-fold radial symmetry that is surrounded by microtubule (MT) walls (as observed by EM, see Figure 1A) [1]. They are found in most eukaryotic cells and form two important structures — cilia and centrosomes. Cilia are formed when the centriole migrates to the plasma membrane and generates a MT-based protrusion that can be either motile or non-motile. When motile, cilia can either be involved in the propulsion of the cell itself or cause the movement of air/liquids across its surface. When non-motile, cilia form the basis of a specialised membrane domain where signalling molecules are concentrated. Centrioles can also form centrosomes, the main microtubule organising centres of the cell, and play an important part in organising the poles of the mitotic spindle [2,3].

In light of these important roles, it is not surprising that centriole dysfunction is of medical importance. Disruption of centriole formation or function can result in malfunctioning cilia, leading to a variety of human pathologies commonly known as ‘ciliopathies' [3,4]. Meanwhile, defects in centrosome function can give rise to microcephaly, a condition characterised by an abnormally small brain [5,6]. In addition, centrosome defects can lead to problems in mitotic spindle formation and alignment. As a result, centrosome malfunction has been associated with several types of cancer [7,8].

Centrosomes are composed of a centriole surrounded by a cloud of proteinaceous material known as the pericentriolar material (PCM) [9]. The PCM, nucleated from around the older ‘mother’ centriole (see below), appears unstructured by EM, but super-resolution microscopy reveals higher order organisational features [10–13]. The exact nature of the PCM remains controversial, and there is evidence supporting both a liquid condensate and/or a solid scaffold structure [14–16]. Regardless, the PCM provides a site for the nucleation of MTs, as well as a site for the concentration of many signalling molecules [17].

Centrosomes are dynamic structures that change throughout the cell cycle. At the beginning of a cycle, each centrosome contains one centriole, from which a ‘daughter' centriole grows in an orthogonal orientation during S phase. At the end of the cell cycle the centriole pair separates, and the daughter centriole is able to mature and nucleate its own daughter and PCM [18]. The size of the PCM and its ability to recruit MTs also changes. Throughout much of the cell cycle centrosomes present very little PCM. In preparation for mitosis, the PCM dramatically expands in size, in a process termed ‘centrosome maturation’ [19]. This prepares the organelle to nucleate the increased number of MTs required for spindle assembly.

The general morphology of centrioles, such as their 9-fold radial symmetry and the presence of MT walls, is well conserved throughout the evolution of eukaryotes, although some organisms have subsequently lost their ability to form centrioles, centrosomes or both [20]. However, the exact structure (e.g. length, composition, etc) can vary between species and even between cells within a single organism [21].

Over the years, both genetic and biochemical studies have identified the key components required for centriole assembly and PCM formation. Although several hundred proteins localise to the centrosome [22], only a handful are essential for centriole assembly (Plk4, Sas-6, Ana2, Sas-4 and Asl) and centrosome formation (Polo, Cnn and Spd-2) [3,23–27]. The fly pathway is summarised in Figure 1B with *Drosophila* names given hereon. Both the molecular players and the pathway that drives centriole formation appear to be remarkably well conserved (human pathway is shown in Figure 1C). Although great strides have been made, the exact function of these key components, and how they interact in both space and time to form the centrosome, is still unclear.

## Fruit fly embryos as a model to study centrioles and centrosomes

The embryo of the fruit fly *Drosophila melanogaster* is an excellent system for studying centrosome and centriole dynamics. During the first three hours of development, the fruit fly embryo is a syncytium, where centrosomes grow and divide rapidly and in synchrony, as successive cell cycles take place within a common cytoplasm [28,29] . This enables many dynamic events, such as the growth of the cartwheel, duplication of centrioles and PCM growth, to be measured relatively quickly and in large numbers, leading to less experimental noise and hence the ability to detect more subtle effects [30–34]. This is also facilitated, from an imaging perspective, by the preferential localisation of centrioles and centrosomes to the cortex of the embryo, where they are in close proximity to the coverslip. There are also other advantages. In fruit flies, the very first centriole of the developing embryo is brought in by the sperm upon fertilisation. As such, unfertilised *Drosophila* eggs (which females naturally lay, facilitating their collection) provide a powerful control system, where no centrioles or centrosomes exist, but which contain all necessary components to form these structures [35–37].

These advantages place *Drosophila* embryos at the forefront of centriole and centrosome quantitative imaging, and hence the dynamic study of these organelles. *Drosophila* embryo centrioles also lack additional structural features (such as appendages) and other protein components found in higher organisms. They therefore represent a simpler form of this organelle that contains only its fundamental, conserved components and lacks the redundancies and complexities of other systems.

Despite these advantages, one particular issue has limited the study of the pathway of centriole and centrosome assembly in *Drosophila*. Knock out mutations of any of the five core centriole assembly factors (Plk4, Sas-6, Ana2, Sas-4, Asl) leads to the absence of centrioles [32,35,38–40]. This complete absence makes it hard to tease apart the interaction between these essential proteins during centriole formation. As centrosomal PCM formation requires the presence of a centriole around which to form, this also frustrates investigations to identify their precise role in guiding PCM assembly.

We recently published a paper presenting an experimental model that allows us to study the relationship between these core centriole duplication factors and their role in PCM recruitment in the absence of core factors in the fruit fly embryo [41]. Here, I discuss in some detail how Sas-6 Ana2 Particles (SAPs) resemble (and differ) from centrioles, and how they can be used to probe specific questions in centriole and centrosome biology. I also place them in the wider context of the various models available in the field.

## Characterising SAPs

SAPs were first observed when our group mildly co-over-expressed both Sas-6 and Ana2, two of the five core centriole duplication proteins, in the *Drosophila* egg [42]. As mentioned above, these eggs are unfertilised, and hence do not contain centrioles. Yet, we could observe the formation of ‘particles’ in the cytoplasm, which were not present when either Sas-6 or Ana2 were over-expressed individually. This suggested that Sas-6 and Ana2 are able to co-assemble into higher order structures in unfertilised eggs.

Further investigation allowed us to characterise these structures in more detail [41]. We found that SAPs are similar to bona fide centrioles in several ways. First, when SAPs were taken from unfertilised eggs and injected into developing embryos (which are fertilised, and hence have hundreds of centrosomes duplicating every cell cycle), they were able to organise robust MT asters whose dynamics cycled in synchrony with the MTs nucleated from endogenous centrosomes. This intriguing result suggested that SAPs can recruit MT nucleation factors in a manner indistinguishable from the recruitment by centrosomes, and that this recruitment is subject to the same cell-cycle regulation cues.

We therefore investigated whether SAPs were able to recruit other centriole and PCM components, and whether this organisation mimicked that seen in bona fide centrosomes. We showed by super-resolution microscopy that this is indeed the case.

In cycling *Drosophila* embryos, recruitment and scaffold formation of the PCM components Spd-2 and Cnn is dependent on their phosphorylation, with the cytoplasmic pool of these proteins remaining unphosphorylated [43–45]. We showed that both Spd-2 and Cnn recruited by SAPs contained similar phosphorylation modifications to those observed in centrosomes, suggesting that the recruitment of a PCM scaffold by SAPs mimics that of bona fide centrioles.

## SAPs: not just non-specific aggregates

Our initial characterisation suggested that SAPs are able to act as a platform for centriole and PCM component recruitment. But are they just non-specific aggregates or do these structures rely on specific, and biologically relevant, interactions between Sas-6 and Ana2 proteins?

Sas-6 is one of the five essential centriole duplication proteins, and the major component of the characteristic 9-fold symmetric cartwheel structure at the centre of centrioles (Figure 1A). Crystal structures of Sas-6 from various species (including fruit flies) have been solved, providing an insight to the role of this protein [46–49]. Nine Sas-6 dimers multimerise through specific interactions in their N-terminal headgroups to form the central hub. Meanwhile, the extended coiled coil and C-terminus of each dimer gives rise to the spokes of the cartwheel. Point mutations in conserved headgroup residues (e.g. *Drosophila* F143 → D) disrupts essential interactions and prevents cartwheel and centriole formation [46,48,49]. We found that such mutant forms of Sas-6 were unable to form SAPs when over-expressed in combination with wild type Ana2.

Similarly, Ana2 contains several conserved domains: a C-terminal STAN domain; a central coiled-coil region; and a short N-terminus region [42,48,50]. Crystal structures have shown that Ana2 can homo-oligomerise via the central coiled-coil region, and that this oligomerisation is essential for centriole formation [48,51]. SAP formation was also prevented when a form of Ana2 lacking this coiled-coil domain was co-over-expressed with Sas-6.

The interaction between Sas-6 and Ana2 is dependent on the STAN domain located in the C-terminus of Ana2 [52–54], although the exact position of binding within Sas-6 is still unclear [55]. This interaction is driven by the phosphorylation of the STAN domain [52–54]. SAPs failed to form when we co-over-expressed Sas-6 and a mutant form of Ana2 lacking the STAN domain. Expression of phospho-null versions that inhibit Ana2 binding to Sas-6 reduced, although did not completely prevent, SAP formation [52–54]. In endogenous centriole formation, it is thought that the kinase Plk4 is responsible for the phosphorylation of the STAN domain [52–54]. Surprisingly, SAPs still form in the absence of Plk4, suggesting that, at least in the context of SAP formation, a second redundant kinase maybe be responsible for this phosphorylation.

Together, this set of experiments led us to conclude that SAP formation relies on many of the molecular interactions between Sas-6 and Ana2 and their regulators that are known to be necessary for bona fide centriole formation. We are therefore confident that they are not simply non-specific aggregates of Sas-6 and Ana2 molecules.

## SAPs: a ‘useful artefact’

Although our observations show that SAPs share many properties with centrioles, it is also clear that they are not centrioles. SAPs are much larger, have higher levels of Sas-6 and Ana2, and appear to lack any observable cartwheel structure by EM. The lack of a cartwheel structure, however, may be the reason why these SAPs can form even in eggs that lack certain essential centriole assembly factors. We discuss one example here of how this has allowed us to investigate the pathway of SAP assembly.

SAPs can form in eggs that lack the core centriole duplication factor Asterless (Asl) [41]. Whilst the role of Asl in centriole duplication has been well established [56], there is both evidence for [57–59] and against [40,60] a role for Asl in PCM formation. This observation provided a unique opportunity to examine whether PCM could form in the absence of Asl.

*Drosophila* genome-wide RNAi screens for genes involved in mitotic PCM formation found three significant hits: Polo, Spd-2 and Cnn (RNAi to Asl in these screens resulted in an absence of centrioles, and hence its role in PCM formation could not be established) [61–63]. In *Drosophila* embryos, Spd-2 is recruited to the centriole wall and fluxes outwards, forming a scaffold [34]. Polo kinase can then bind to this scaffold and phosphorylate Cnn [12,44]. This phosphorylation then causes Cnn to form a scaffold [43,45]. The Spd-2/Cnn scaffold provides the platform that subsequently recruits other PCM components, including those required for MT nucleation. The exact nature of Spd-2 recruitment to the centriole wall is unclear, although previous evidence strongly points to a role for Asl [59].

In eggs expressing a truncated form of Asl (which supports centriole duplication, but not PCM formation, in other *Drosophila* tissues [40,58]), PCM recruitment to SAPs is blocked. Surprisingly, in the complete absence of Asl, PCM could still form around SAPs (although less efficiently than around SAPs in WT eggs). This suggests that, in normal conditions, Asl plays a role in PCM formation but, in the total absence of this protein, an alternative pathway is present that can recruit PCM, albeit less efficiently. This alternative PCM formation pathway is dependent on Spd-2, as SAPs lacking both Asl and Spd-2 form no PCM.

Using SAPs, we have confirmed a role for Asl in PCM recruitment, as well as demonstrated the existence of an alternative PCM recruitment pathway that primarily relies on Spd-2. This is only one example of how SAPs could be used to probe outstanding questions regarding the role of essential centriole duplication proteins, and we direct you to our paper for further examples [41].

## SAPs in *Drosophila* eggs: just one of many models

Despite their many similarities, SAPs are not centrioles. This can be advantageous, enabling experiments and assays not possible with bona fide centrioles [41]. Although the organisation of proteins around SAPs mimics those around the centriole wall, the exact behaviour, organisation and function of these proteins may not be identical in the two structures. In addition, many of our observations in SAPs have taken place in unfertilised eggs, where standard cell cycle cues are not present. Therefore, and although they are a useful model, any conclusions with regards to how events are regulated must be taken with caution.

Other *in vivo Drosophila* approaches have been used over the years to probe the role of core centriole duplication factors in PCM formation. The use of hypomorphic alleles has helped to identify potential roles for these proteins (*asl[1]* hypomorph) [40,58]. The injection of antibodies that inhibit the function of core centriole duplication factor proteins into cycling WT embryos has provided further insight into the relationship between core centriole proteins and the PCM [33,59,64]. Similarly, the injection of mRNA into embryos or transgenic lines encoding mutated forms of centriole duplication factors have also been used to identify important residues and domains [56,65].

The role of core centriole factors has also been investigated in other tissues, such as the male germline of *Drosophila*. A useful genetic trick can be used by looking at spermatocyte cells that are homozygous for a centriole duplication mutation but which derive from heterozygous mothers. When the male germ cells are forming in this embryo, there is still enough wild type protein in the cytoplasm (through maternal contribution) to allow the formation of a few centrioles. Further in development the wild type protein is completely depleted, allowing us to examine ‘wild type’ centrioles in a ‘mutant' cytoplasmic context [40,60,66,67]. In such a system it is only possible to study the role of proteins that exchange actively with the cytoplasm rather than those that were incorporated into centrioles during their formation.

Although all these approaches can provide insights into the role of core centriole proteins, none of them categorically assesses the effect of complete protein absence. Rather, they reduce protein levels/function, and therefore residual protein may obscure certain phenotypes; or they use centrioles that were formed in the presence of these proteins. In such cases, the advantages of a complementary analysis using SAPs in a complete mutant background is apparent, as illustrated in the case of Asl.

SAPs in *Drosophila* eggs may not be the only ‘SAP-like' model available in the toolbox of the centriole/centrosome biologist. Previous observations from our lab shows that the co-over-expression of Sas-6 and Ana2 in *Drosophila* spermatocytes also generates aggregates in this tissue. Interestingly, these ‘testes SAPs' do not nucleate PCM or MTs but instead display an ultrastructure strikingly similar to the centriole cartwheel [68]. This suggests that these SAPs may be useful to probe the structural role of centriole duplication proteins in a way that ‘egg SAPs' cannot. Intriguingly, similar structures are seen in *Drosophila* spermatocytes when Sas-6 and another centriole duplication factor, Cep135/Bld10, are co-over-expressed, suggesting that other proteins may be involved in forming these aggregates [69].

Fascinatingly, the formation of SAP-like structures does not seem to be unique to *Drosophila*. For example, the expression of Plk4 can give rise to aggregates that are able to organise centriole and PCM factors and nucleate MTs, not unlike our observations, in *Xenopus* cells [70]. Similarly, ectopic expression of Cep63 and Cep152 (the human homologue of Asl) as well as Plk4 in human cells gives rise to similar aggregates [71,72]. This suggests that this useful, self-organising capacity of centriole proteins might be a wider phenomenon that can be exploited for investigations in other model systems, no doubt with unique features that will allow other aspects of centriole and centrosome biology to be explored.

## Perspectives

- Centrioles are cellular organelles of medical importance, and their dysfunction may result in ciliopathies, microcephaly or cancer. Yet, a full understanding of how their molecular components interact is hampered by the total absence of these structures when core components are absent.
- Sas-6 Ana2 particles (SAPs) are an experimental model that overcomes these limitations and is allowing us to probe at important molecular interactions in these organelles.
- The self-organising capacity of centriole proteins demonstrated by SAPs may be a wider phenomenon that can be exploited for investigations of centriole biology in other model systems.

## Open Access

Open access for this article was enabled by the participation of University of Oxford in an all-inclusive *Read & Publish* pilot with Portland Press and the Biochemical Society under a transformative agreement with JISC.

## Abbreviations

MT: microtubule
PCM: pericentriolar material
SAPs: Sas-6 Ana2 Particles

## References

[1] LeGuennec, M., Klena, N., Aeschlimann, G., Hamel, V. and Guichard, P. (2020) Overview of the centriole architecture. Curr. Opin. Struct. Biol. 66, 58–65 10.1016/j.sbi.2020.09.015 33176264

[2] Nigg, E.A. and Raff, J.W. (2009) Centrioles, centrosomes, and cilia in health and disease. Cell 139, 663–678 10.1016/j.cell.2009.10.036 19914163

[3] Nigg, E.A. and Holland, A.J. (2018) Once and only once: mechanisms of centriole duplication and their deregulation in disease. Nat. Rev. Mol. Cell Biol. 19, 297–312 10.1038/nrm.2017.127 29363672 PMC5969912

[4] Bettencourt-Dias, M., Hildebrandt, F., Pellman, D., Woods, G. and Godinho, S.A. (2011) Centrosomes and cilia in human disease. Trends Genet. 27, 307–315 10.1016/j.tig.2011.05.004 21680046 PMC3144269

[5] Jayaraman, D., Bae, B.-I. and Walsh, C.A. (2018) The genetics of primary microcephaly. Annu. Rev. Genom. Hum. Genet. 19, 177–200 10.1146/annurev-genom-083117-021441 29799801

[6] Nano, M. and Basto, R. (2017) Consequences of centrosome dysfunction during brain development. Adv. Exp. Med. Biol. 1002, 19–45 10.1007/978-3-319-57127-0_2 28600781

[7] Goundiam, O. and Basto, R. (2020) Centrosomes in disease: how the same music can sound so different? Curr. Opin. Struct. Biol. 66, 74–82 10.1016/j.sbi.2020.09.011 33186811

[8] Chan, J.Y. (2011) A clinical overview of centrosome amplification in human cancers. Int. J. Biol. Sci. 7, 1122–1144 10.7150/ijbs.7.1122 22043171 PMC3204404

[9] Pimenta-Marques, A. and Bettencourt-Dias, M. (2020) Pericentriolar material. Curr. Biol. 30, R687–R689 10.1016/j.cub.2020.04.064 32574625

[10] Mennella, V., Keszthelyi, B., McDonald, K.L., Chhun, B., Kan, F., Rogers, G.C. et al. (2012) Subdiffraction-resolution fluorescence microscopy reveals a domain of the centrosome critical for pericentriolar material organization. Nat. Cell Biol. 14, 1159–1168 10.1038/ncb2597 23086239 PMC3767400

[11] Sonnen, K.F., Schermelleh, L., Leonhardt, H. and Nigg, E.A. (2012) 3D-structured illumination microscopy provides novel insight into architecture of human centrosomes. Biol Open. 1, 965–976 10.1242/bio.20122337 23213374 PMC3507176

[12] Fu, J. and Glover, D.M. (2012) Structured illumination of the interface between centriole and peri-centriolar material. Open Biol. 2, 120104 10.1098/rsob.120104 22977736 PMC3438536

[13] Lawo, S., Hasegan, M., Gupta, G.D. and Pelletier, L. (2012) Subdiffraction imaging of centrosomes reveals higher-order organizational features of pericentriolar material. Nat. Cell Biol. 14, 1148–1158 10.1038/ncb2591 23086237

[14] Woodruff, J.B., Wueseke, O. and Hyman, A.A. (2014) Pericentriolar material structure and dynamics. Philos. Trans. R. Soc. Lond., B Biol. Sci. 369, 20130459 10.1098/rstb.2013.0459 25047613 PMC4113103

[15] Woodruff, J.B. (2020) The material state of centrosomes: lattice, liquid, or gel? Curr. Opin. Struct. Biol. 66, 139–147 10.1016/j.sbi.2020.10.001 33248427

[16] Raff, J.W. (2019) Phase separation and the centrosome: a fait accompli? Trends Cell Biol. 29, 612–622 10.1016/j.tcb.2019.04.001 31076235

[17] Wu, J. and Akhmanova, A. (2017) Microtubule-Organizing centers. Annu. Rev. Cell Dev. Biol. 33, 51–75 10.1146/annurev-cellbio-100616-060615 28645217

[18] Fu, J., Lipinszki, Z., Rangone, H., Min, M., Mykura, C., Chao-Chu, J. et al. (2016) Conserved molecular interactions in centriole-to-centrosome conversion. Nat. Cell Biol. 18, 87–99 10.1038/ncb3274 26595382 PMC4719191

[19] Palazzo, R.E., Vogel, J.M., Schnackenberg, B.J., Hull, D.R. and Wu, X. (2000) Centrosome maturation. Curr. Top. Dev. Biol. 49, 449–470 10.1016/S0070-2153(99)49021-0 11005031

[20] Carvalho-Santos, Z., Azimzadeh, J., Pereira-Leal, J.B. and Bettencourt-Dias, M. (2011) Evolution: tracing the origins of centrioles, cilia, and flagella. J. Cell Biol. 194, 165–175 10.1083/jcb.201011152 21788366 PMC3144413

[21] Gupta, A. and Kitagawa, D. (2018) Ultrastructural diversity between centrioles of eukaryotes. J. Biochem. 164, 1–8 10.1093/jb/mvy031 29462371

[22] Alves-Cruzeiro, J.M.D.C., Alves-Cruzeiro, J.M.D.C., Nogales-Cadenas, R., Nogales-Cadenas, R., Pascual-Montano, A.D. and Pascual-Montano, A.D. (2014) CentrosomeDB: a new generation of the centrosomal proteins database for human and *Drosophila melanogaster*. Nucleic Acids Res. 42, D430–D436 10.1093/nar/gkt1126 24270791 PMC3964966

[23] Banterle, N. and Gönczy, P. (2017) Centriole biogenesis: from identifying the characters to understanding the plot. Annu. Rev. Cell Dev. Biol. 33, 23–49 10.1146/annurev-cellbio-100616-060454 28813178

[24] Gönczy, P. and Hatzopoulos, G.N. (2019) Centriole assembly at a glance. J. Cell Sci. 132, jcs228833 10.1242/jcs.228833 30787112

[25] Lattao, R., Kovacs, L. and Glover, D.M. (2017) The centrioles, centrosomes, basal bodies, and cilia of drosophila melanogaster. Genetics 206, 33–53 10.1534/genetics.116.198168 28476861 PMC5419478

[26] Loncarek, J. and Bettencourt-Dias, M. (2018) Building the right centriole for each cell type. J. Cell Biol. 217, 823–835 10.1083/jcb.201704093 29284667 PMC5839779

[27] Conduit, P.T., Wainman, A. and Raff, J.W. (2015) Centrosome function and assembly in animal cells. Nat. Rev. Mol. Cell Biol. 16, 611–624 10.1038/nrm4062 26373263

[28] Foe, V.E. and Alberts, B.M. (1983) Studies of nuclear and cytoplasmic behaviour during the five mitotic cycles that precede gastrulation in drosophila embryogenesis. J. Cell Sci. 61, 31–70 PMID:6411748 10.1242/jcs.61.1.31

[29] Callaini, G. and Riparbelli, M.G. (1990) Centriole and centrosome cycle in the early Drosophila embryo. J. Cell Sci. 97, 539–543 PMID:2127418 10.1242/jcs.97.3.539

[30] Megraw, T.L., Kilaru, S., Turner, F.R. and Kaufman, T.C. (2002) The centrosome is a dynamic structure that ejects PCM flares. J. Cell Sci. 115, 4707–4718 10.1242/jcs.00134 12415014

[31] Lerit, D.A., Jordan, H.A., Poulton, J.S., Fagerstrom, C.J., Galletta, B.J., Peifer, M. et al. (2015) Interphase centrosome organization by the PLP-Cnn scaffold is required for centrosome function. J. Cell Biol. 210, 79–97 10.1083/jcb.201503117 26150390 PMC4494003

[32] Aydogan, M.G., Wainman, A., Saurya, S., Steinacker, T.L., Caballe, A., Novak, Z.A. et al. (2018) A homeostatic clock sets daughter centriole size in flies. J. Cell Biol. 217, 1233–1248 10.1083/jcb.201801014 29500190 PMC5881511

[33] Novak, Z.A., Conduit, P.T., Wainman, A. and Raff, J.W. (2014) Asterless licenses daughter centrioles to duplicate for the first time in Drosophila embryos. Curr. Biol. 24, 1276–1282 10.1016/j.cub.2014.04.023 24835456 PMC4046630

[34] Conduit, P.T., Richens, J.H., Wainman, A., Holder, J., Vicente, C.C., Pratt, M.B. et al. (2014) A molecular mechanism of mitotic centrosome assembly in drosophila. eLife 3, e03399 10.7554/eLife.03399 25149451 PMC4175739

[35] Peel, N., Stevens, N.R., Basto, R. and Raff, J.W. (2007) Overexpressing centriole-replication proteins in vivo induces centriole overduplication and de novo formation. Curr. Biol. 17, 834–843 10.1016/j.cub.2007.04.036 17475495 PMC1885955

[36] Pimenta-Marques, A., Bento, I., Lopes, C.A.M., Duarte, P., Jana, S.C. and Bettencourt-Dias, M. (2016) A mechanism for the elimination of the female gamete centrosome in *drosophila melanogaster*. Science 353, aaf4866 10.1126/science.aaf4866 27229142

[37] Rodrigues-Martins, A., Riparbelli, M., Callaini, G., Glover, D.M. and Bettencourt-Dias, M. (2007) Revisiting the role of the mother centriole in centriole biogenesis. Science 316, 1046–1050 10.1126/science.1142950 17463247

[38] Basto, R., Lau, J., Vinogradova, T., Gardiol, A., Woods, C.G., Khodjakov, A. et al. (2006) Flies without centrioles. Cell 125, 1375–1386 10.1016/j.cell.2006.05.025 16814722

[39] Baumbach, J., Novak, Z.A., Raff, J.W. and Wainman, A. (2015) Dissecting the function and assembly of acentriolar microtubule organizing centers in Drosophila cells in vivo. PLoS Genet. 11, e1005261 10.1371/journal.pgen.1005261 26020779 PMC4447278

[40] Blachon, S., Gopalakrishnan, J., Omori, Y., Polyanovsky, A., Church, A., Nicastro, D. et al. (2008) Drosophila asterless and vertebrate Cep152 Are orthologs essential for centriole duplication. Genetics 180, 2081–2094 10.1534/genetics.108.095141 18854586 PMC2600943

[41] Gartenmann, L., Vicente, C.C., Wainman, A., Novak, Z.A., Sieber, B., Richens, J.H. et al. (2020) Drosophila Sas-6, Ana2 and Sas-4 self-organise into macromolecular structures that can be used to probe centriole and centrosome assembly. J. Cell Sci. 133, jcs244574 10.1242/jcs.244574 32409564 PMC7328145

[42] Stevens, N.R., Dobbelaere, J., Brunk, K., Franz, A. and Raff, J.W. (2010) Drosophila Ana2 is a conserved centriole duplication factor. J. Cell Biol. 188, 313–323 10.1083/jcb.200910016 20123993 PMC2819680

[43] Conduit, P.T., Feng, Z., Richens, J.H., Baumbach, J., Wainman, A., Bakshi, S.D. et al. (2014) The centrosome-specific phosphorylation of Cnn by Polo/Plk1 drives Cnn scaffold assembly and centrosome maturation. Dev. Cell 28, 659–669 10.1016/j.devcel.2014.02.013 24656740 PMC3988887

[44] Alvarez Rodrigo, I., Steinacker, T.L., Saurya, S., Conduit, P.T., Baumbach, J., Novak, Z.A. et al. (2019) Evidence that a positive feedback loop drives centrosome maturation in fly embryos. eLife 8, D430 10.7554/eLife.50130 PMC6733597 31498081

[45] Feng, Z., Caballe, A., Wainman, A., Johnson, S., Haensele, A.F.M., Haensele, A.F.M. et al. (2017) Structural basis for mitotic centrosome assembly in flies. Cell 169, 1078–1089.e13 10.1016/j.cell.2017.05.030 28575671 PMC5457487

[46] van Breugel, M., Hirono, M., Andreeva, A., Yanagisawa, H.-A., Yamaguchi, S., Nakazawa, Y. et al. (2011) Structures of SAS-6 suggest its organization in centrioles. Science 331, 1196–1199 10.1126/science.1199325 21273447

[47] van Breugel, M., Wilcken, R., McLaughlin, S.H., Rutherford, T.J. and Johnson, C.M. (2014) Structure of the SAS-6 cartwheel hub from L 10.7554/eLife.01812 PMC3939493 24596152

[48] Cottee, M.A., Muschalik, N., Johnson, S., Leveson, J., Raff, J.W. and Lea, S.M. (2015) The homo-oligomerisation of both Sas-6 and Ana2 is required for efficient centriole assembly in flies. eLife 4, e07236 10.7554/eLife.07236 26002084 PMC4471874

[49] Kitagawa, D., Vakonakis, I., Olieric, N., Hilbert, M., Keller, D., Olieric, V. et al. (2011) Structural basis of the 9-fold symmetry of centrioles. Cell 144, 364–375 10.1016/j.cell.2011.01.008 21277013 PMC3089914

[50] Cottee, M.A., Muschalik, N., Wong, Y.L., Johnson, C.M., Johnson, S., Andreeva, A. et al. (2013) Crystal structures of the CPAP/STIL complex reveal its role in centriole assembly and human microcephaly. eLife 2, 30 10.7554/eLife.01071 PMC3776556 24052813

[51] David, A., Amartely, H., Rabinowicz, N., Shamir, M., Friedler, A. and Izraeli, S. (2016) Molecular basis of the STIL coiled coil oligomerization explains its requirement for de-novo formation of centrosomes in mammalian cells. Sci. Rep. 6, 24296 10.1038/srep24296 27075531 PMC4830966

[52] Dzhindzhev, N.S., Tzolovsky, G., Lipinszki, Z., Schneider, S., Lattao, R., Fu, J. et al. (2014) Plk4 phosphorylates Ana2 to trigger Sas6 recruitment and procentriole formation. Curr. Biol. 24, 2526–2532 10.1016/j.cub.2014.08.061 25264260 PMC4229625

[53] Ohta, M., Ashikawa, T., Nozaki, Y., Kozuka-Hata, H., Goto, H., Inagaki, M. et al. (2014) Direct interaction of Plk4 with STIL ensures formation of a single procentriole per parental centriole. Nat. Commun. 5, 5267 10.1038/ncomms6267 25342035 PMC4220463

[54] Kratz, A.-S., Kratz, A.S., Barenz, F., Bärenz, F., Richter, K.T., Richter, K.T. et al. (2015) Plk4-dependent phosphorylation of STIL is required for centriole duplication. Biol. Open 4, 370–377 10.1242/bio.201411023 25701666 PMC4359743

[55] Fatalska, A., Dzhindzhev, N.S., Dadlez, M. and Glover, D.M. (2020) Interaction interface in the C-terminal parts of centriole proteins Sas6 and Ana2. Open Biol. 10, 200221 10.1098/rsob.200221 33171067 PMC7729032

[56] Dzhindzhev, N.S., Yu, Q.D., Weiskopf, K., Tzolovsky, G., Cunha-Ferreira, I., Riparbelli, M. et al. (2010) Asterless is a scaffold for the onset of centriole assembly. Nature 467, 714–718 10.1038/nature09445 20852615

[57] Bonaccorsi, S., Giansanti, M.G. and Gatti, M. (1998) Spindle self-organization and cytokinesis during male meiosis in asterless mutants of *Drosophila melanogaster*. J. Cell Biol. 142, 751–761 10.1083/jcb.142.3.751 9700163 PMC2148166

[58] Varmark, H., Llamazares, S., Rebollo, E., Lange, B., Reina, J., Schwarz, H. et al. (2007) Asterless is a centriolar protein required for centrosome function and embryo development in Drosophila. Curr. Biol. 17, 1735–1745 10.1016/j.cub.2007.09.031 17935995

[59] Conduit, P.T., Brunk, K., Dobbelaere, J., Dix, C.I., Lucas, E.P. and Raff, J.W. (2010) Centrioles regulate centrosome size by controlling the rate of Cnn incorporation into the PCM. Curr. Biol. 20, 2178–2186 10.1016/j.cub.2010.11.011 21145741

[60] Galletta, B.J., Jacobs, K.C., Fagerstrom, C.J. and Rusan, N.M. (2016) Asterless is required for centriole length control and sperm development. J. Cell Biol. 213, 435–450 10.1083/jcb.201501120 27185836 PMC4878089

[61] Bettencourt-Dias, M., Giet, R., Sinka, R., Mazumdar, A., Lock, W.G., Balloux, F. et al. (2004) Genome-wide survey of protein kinases required for cell cycle progression. Nature 432, 980–987 10.1038/nature03160 15616552

[62] Dobbelaere, J., Josué, F., Suijkerbuijk, S., Baum, B., Tapon, N. and Raff, J. (2008) A genome-wide RNAi screen to dissect centriole duplication and centrosome maturation in drosophila. PLoS Biol. 6, e224 10.1371/journal.pbio.0060224 18798690 PMC2535660

[63] Goshima, G., Wollman, R., Goodwin, S.S., Zhang, N., Scholey, J.M., Vale, R.D. et al. (2007) Genes required for mitotic spindle assembly in Drosophila S2 cells. Science 316, 417–421 10.1126/science.1141314 17412918 PMC2837481

[64] Ramani, A., Mariappan, A., Gottardo, M., Mandad, S., Urlaub, H., Avidor-Reiss, T. et al. (2018) Plk1/Polo phosphorylates Sas-4 at the onset of mitosis for an efficient recruitment of pericentriolar material to centrosomes. Cell Rep. 25, 3618–3630.e6 10.1016/j.celrep.2018.11.102 30590037

[65] Novak, Z.A., Wainman, A., Gartenmann, L. and Raff, J.W. (2016) Cdk1 phosphorylates drosophila Sas-4 to recruit polo to daughter centrioles and convert them to centrosomes. Dev. Cell 37, 545–557 10.1016/j.devcel.2016.05.022 27326932 PMC4918730

[66] Riparbelli, M.G. and Callaini, G. (2011) Male gametogenesis without centrioles. Dev. Biol. 349, 427–439 10.1016/j.ydbio.2010.10.021 20974123

[67] Blachon, S., Cai, X., Roberts, K.A., Yang, K., Polyanovsky, A., Church, A. et al. (2009) A proximal centriole-like structure is present in Drosophila spermatids and can serve as a model to study centriole duplication. Genetics 182, 133–144 10.1534/genetics.109.101709 19293139 PMC2674812

[68] Stevens, N.R., Roque, H. and Raff, J.W. (2010) DSas-6 and Ana2 coassemble into tubules to promote centriole duplication and engagement. Dev. Cell 19, 913–919 10.1016/j.devcel.2010.11.010 21145506 PMC4159445

[69] Jo, K.H., Jaiswal, A., Khanal, S., Fishman, E.L., Curry, A.N. and Avidor-Reiss, T. (2019) Poc1b and Sas-6 function together during the atypical centriole formation in *Drosophila melanogaster*. Cells 8, 841 10.3390/cells8080841 PMC6721650 31387336

[70] Montenegro Gouveia, S., Zitouni, S., Kong, D., Duarte, P., Ferreira Gomes, B., Sousa, A.L. et al. (2018) PLK4 is a microtubule-associated protein that self-assembles promoting de novoMTOC formation. J. Cell Sci. 132, jcs219501 10.1242/jcs.219501 30237222 PMC6398482

[71] Ahn, J.I., Park, J.-E., Meng, L., Zhang, L., Kim, T.-S., Kruhlak, M.J. et al. (2020) Phase separation of the Cep63•Cep152 complex underlies the formation of dynamic supramolecular self-assemblies at human centrosomes. Cell Cycle 19, 3437–3457 10.1080/15384101.2020.1843777 33208041 PMC7781590

[72] Park, J.-E., Zhang, L., Bang, J.K., Andresson, T., DiMaio, F. and Lee, K.S. (2019) Phase separation of Polo-like kinase 4 by autoactivation and clustering drives centriole biogenesis. Nat. Commun. 10, 23–19 10.1038/s41467-018-07919-y 31672968 PMC6823436

